# Diagnostic performance of AI-powered prostate MRI against biopsy ground truth: quasi-continuous risk scoring versus fixed PI-RADS thresholds

**DOI:** 10.1186/s40644-026-01126-5

**Published:** 2026-09-15

**Authors:** Nadine Bayerl, Joel Appel, Maximilian Schmidt, Alexander Cavallaro, Robert Grimm, Heinrich von Busch, Bernd Wullich, Arndt Hartmann, Michael Schlicht, Michael Uder, Matthias S. May, Stephan Ellmann

**Affiliations:** 1 Institute of Radiology, Friedrich-Alexander-Universität Erlangen-Nürnberg and Uniklinikum Erlangen, Maximiliansplatz 3, 91054 Erlangen, Germany; 2 Research & Clinical Translation, Magnetic Resonance, Siemens Healthineers AG, Allee am Röthelheimpark 2, 91052 Erlangen, Germany; 3 Digital & Automation, Siemens Healthineers AG, Siemensstraße 3, 91301 Forchheim, Germany; 4 Clinic of Urology and Pediatric Urology, Friedrich-Alexander-Universität Erlangen-Nürnberg and Uniklinikum Erlangen, Maximiliansplatz 1, 91054 Erlangen, Germany; 5 Comprehensive Cancer Center Erlangen-EMN (CCC ER-EMN), Östliche Stadtmauerstraße 30, 91054 Erlangen, Germany; 6 Bavarian Cancer Research Center (BZKF), Östliche Stadtmauerstraße 30, 91054 Erlangen, Germany; 7 Institute of Pathology, Friedrich-Alexander-Universität Erlangen-Nürnberg and Uniklinikum Erlangen, Krankenhausstr. 8-10, 91054 Erlangen, Germany; 8 Clinic of Internal Medicine III, Klinikum Bamberg, Buger Straße 80, 96050 Bamberg, Germany; 9 Imaging Science Institute (ISI) Erlangen, Ulmenweg 18, 91054 Erlangen, Germany; 10 Radiologisch-Nuklearmedizinisches Zentrum (RNZ.), Martin-Richter-Straße 43, 90489 Nürnberg, Germany

**Keywords:** Magnetic resonance imaging, Prostatic neoplasms, Decision support systems, clinical, Diagnosis, computer-assisted, Deep learning, Early detection of cancer

## Abstract

**Background:**

Multiparametric prostate MRI is central to prostate cancer (PCa) diagnostics, yet PI-RADS interpretation suffers from inter-reader variability. This study aimed to evaluate a commercial AI algorithm for cancer detection in prostate MRI against histopathology, comparing its standard PI-RADS classification with its quasi-continuous Level-of-Suspicion (LoS) score as decision variables.

**Methods:**

In this retrospective single-center study, 122 mpMRI examinations, each followed by systematic and/or fusion-targeted biopsy, were analyzed. Histopathology served as the reference standard for any PCa (Gleason ≥ 6) and clinically significant PCa (csPCa; Gleason ≥ 7). Diagnostic performance of the algorithm’s PI-RADS and LoS outputs was assessed by ROC analysis. AUCs were compared using DeLong’s test. The Youden-optimal LoS cutoff was determined, its optimism was quantified by bootstrap internal validation, and the calibration of the LoS score was assessed after logistic recalibration.

**Results:**

For csPCa, PI-RADS yielded an AUC of 0.833 (95% CI: 0.764–0.901), versus 0.882 (95% CI: 0.820–0.943) for the LoS score (*p* = 0.106). At PI-RADS ≥ 4, sensitivity was 94.7% and specificity 63.1%. The Youden-optimal LoS cutoff was 81. At this exploratory threshold, sensitivity remained 94.7% while specificity was 70.8%, with positive and negative predictive values of 74.0% and 93.9%, respectively. Bootstrap internal validation indicated modest optimism, with corrected estimates of 92.9% for sensitivity and 69.1% for specificity. After logistic recalibration, the bootstrap-corrected calibration slope was 0.98 with an intercept of -0.01.

**Conclusion:**

In this retrospective single-center cohort, the AI algorithm achieved high diagnostic accuracy for csPCa. Its quasi-continuous LoS score provided an operating point combining high sensitivity with numerically higher specificity than the PI-RADS ≥ 4 threshold. Neither this difference (exact McNemar *p* = 0.063) nor the difference in AUC between the two classifiers (*p* = 0.106) reached statistical significance, so the finding should be regarded as a promising trend rather than a demonstrated advantage. Prospective confirmation is required before AI-assisted standardized interpretation is integrated into prostate MRI workflows on this basis.

## Background

Prostate cancer (PCa) remains one of the most frequently diagnosed malignancies in men worldwide [1]. The diagnostic pathway for PCa has been transformed by multiparametric magnetic resonance imaging (mpMRI), which enables lesion localization, improves risk stratification, and facilitates targeted biopsies. This approach has led to a reduction in unnecessary procedures and has decreased the rate of pathological upgrading at radical prostatectomy compared to systematic sampling alone [2–4]. To standardize image acquisition, interpretation, and reporting, the Prostate Imaging-Reporting and Data System (PI-RADS) was established, with its current version being v2.1 [5, 6]. However, despite this standardization, significant inter- and intra-reader variability persists, remaining a key barrier to uniform quality of care [7, 8].

To address these challenges, artificial intelligence (AI) systems, particularly those based on deep learning, have emerged as powerful decision support tools [9]. AI can enforce the consistent application of diagnostic criteria, stabilize performance, and help scale expertise beyond specialized centers [10]. Many studies leverage biparametric MRI (bpMRI) inputs to reduce scan times and avoid gadolinium-based contrast agents without compromising diagnostic accuracy [11, 12]. Clinical evaluations of commercial AI tools have demonstrated diagnostic performance comparable to meta-analytic benchmarks for human readers with AI’s potential to rule out disease at low PI-RADS categories while maintaining high accuracy for clinically significant PCa (csPCa) at higher thresholds [13]. While many AI tools reproduce the familiar ordinal PI-RADS framework, the commercially available AI-Rad Companion (AIRC) Prostate MR software (Siemens Healthineers AG, Forchheim, Germany) additionally provides a Level-of-Suspicion (LoS) output in its recent versions - a more detailed, model-derived score. This quasi-continuous output can support threshold optimization for specific clinical needs, such as maximizing sensitivity for ruling out csPCa or prioritizing high specificity where biopsy resources are constrained [14–16]. The LoS score may capture information beyond the ordinal nature of categorical PI-RADS thresholds, allowing more refined operating points and a more transparent trade-off between sensitivity and specificity.

Therefore, we performed a clinical evaluation of this AI algorithm for prostate MRI which outputs both a PI-RADS category and a quasi-continuous LoS score for clinical decision support. The objectives of this study were: (1) to quantify the diagnostic performance for detecting (a) any PCa (Gleason ≥ 6) and (b) csPCa (Gleason ≥ 7) using the algorithm’s standard PI-RADS classification at conventional cutoffs, (2) to evaluate the quasi-continuous LoS score as a decision variable by identifying a Youden-optimal operating point and reporting its associated diagnostic metrics, and (3) to contextualize the algorithm’s performance by: (a) directly comparing the diagnostic accuracy of the LoS and PI-RADS outputs, and (b) descriptively contextualizing the optimal LoS operating point against contemporary, large-scale meta-analytic reference estimates for expert human readers. The primary hypothesis was that the quasi-continuous LoS score would demonstrate superior diagnostic accuracy compared to the algorithm’s ordinal PI-RADS classification. The comparison with published meta-analytic benchmarks is presented as a descriptive contextualization rather than as a formal hypothesis test.

## Methods

### Study design and population

This retrospective, single-center study adhered to the Declaration of Helsinki and received ethics approval with a waiver of informed consent from the Ethics Committee of the Friedrich-Alexander-Universität Erlangen-Nürnberg (approval number: 260_19Bc). We included 123 consecutive prostate mpMRI examinations performed for suspected prostate cancer between June 2014 and July 2017. All patients subsequently underwent biopsy within one month of the MRI examination to establish the definitive diagnosis. In accordance with institutional routine, the biopsy procedure for patients with an MRI-visible lesion consisted of both targeted cores via MRI-ultrasound fusion and a standard 12-core systematic biopsy. Patients without an identifiable target on MRI underwent a systematic biopsy alone. The histopathological analysis of all biopsy cores served as the reference standard for defining (a) any PCa (Gleason score ≥ 6) and (b) csPCa (Gleason score ≥ 7). We excluded one patient because one of the required input series (diffusion weighted imaging - DWI) was missing. Thus, the finally investigated dataset comprised 122 mpMRI examinations. Each examination was paired with its own biopsy. Serum PSA obtained before biopsy was recorded for all examinations.

### MRI acquisition

MRI was performed at 1.5 T (MAGNETOM Aera, *n* = 7) and 3 T scanners (MAGNETOM Skyra, *n* = 80; MAGNETOM Verio, *n* = 32; MAGNETOM Vida, *n* = 3; all, Siemens Healthineers AG, Erlangen, Germany) using pelvic phased-array coils. Protocols comprised high-resolution T2-weighted imaging and diffusion-weighted imaging (with ADC maps), consistent with international PI-RADS guidelines.

### Biopsy and histopathology

All MRI examinations were followed by transrectal or transperineal systematic sampling (typically 12 cores), with additional fusion-targeted cores when an MRI-visible lesion was present, reflecting the current EAU-guideline-recommended reference standard [17]. Biopsies were processed and reported by subspecialized uropathologists. The highest Gleason score across all cores was considered as ground truth.

### AI algorithm and outputs

The evaluated commercial AI implementation was AIRC Prostate MR, version VA60 (Siemens Healthineers AG). The algorithm processed the MRI data in a fully automated way, without user interaction and without access to clinical patient information or histopathological results. It ingests biparametric MRI inputs (T2-weighted and DWI/ADC), first segments the prostate gland, then localizes suspicious lesions, and outputs both (a) a PI-RADS-compatible category and (b) an algorithm-derived LoS score for each detected lesion. The LoS is a quasi-continuous estimate produced by the trained model of the likelihood of the presence of a lesion suspicious of cancer. Detected lesions are assigned an LoS between 60 and 100. An LoS of 60–79 is mapped to an AI-assigned PI-RADS 3, while an LoS of 80–100 results in an AI-assigned PI-RADS 4 or 5, depending on whether the lesion segmentation diameter is smaller or larger/equal to 15 mm, respectively. For this study, the highest PI-RADS and LoS scores for each examination were used. Examinations in which the algorithm detected no lesion were assigned a fixed LoS value of 30 and were therefore counted as negative at every threshold. Where a lesion was detected, LoS values ranged from 60 to 100. No lesion was detected in 31 of the 122 examinations (25.4%).

### Endpoints and statistical analysis

The primary endpoint was the AUC for detecting csPCa (Gleason ≥ 7), computed separately for the PI-RADS classification and the LoS score. Detection of any cancer (Gleason ≥ 6) was analyzed as a secondary endpoint. Further secondary endpoints included sensitivity, specificity, and predictive values at pre-specified PI-RADS cutoffs (≥ 3, ≥ 4, and 5) and at the Youden-optimal LoS threshold, which maximizes the sum of sensitivity and specificity. Because this threshold was both derived and evaluated in the same cohort, its optimism was quantified by bootstrap internal validation with 2,000 resamples. In each resample the Youden-optimal threshold was re-derived and then applied to the original cohort, and the mean difference between resample and original performance was subtracted from the apparent estimates. Calibration of the LoS score was assessed for the csPCa endpoint. Since the LoS is a bounded score rather than a probability, it was first mapped to the probability scale by logistic recalibration. Calibration intercept and slope and the Brier score are reported, with intercept and slope additionally corrected for optimism by bootstrap.

Analyses were conducted in R version 4.4.2 (2024-10-31) (R Foundation for Statistical Computing, Vienna, Austria) using the packages pROC (DeLong’s test, ROC analysis, AUC comparison), epiR (sensitivity, specificity, PPV, NPV with 95% confidence intervals via epi.tests), ggplot2 (visualization), and rms (logistic recalibration and calibration measures). ROC curves were compared using DeLong’s test, the specificities of the two decision rules were compared using the exact McNemar test, and 95% confidence intervals (CI) were calculated. The Area Under the ROC Curve (AUC) was used to estimate diagnostic accuracy. Additional analyses included contingency tables to estimate sensitivity, specificity, positive predictive value (PPV), and negative predictive value (NPV), along with their respective 95% CIs. Published meta-analytic estimates are reported for descriptive context only. No formal statistical comparison against them was performed, because the pooled populations, imaging protocols and clinical settings differ substantially from the present cohort. For all tests, p-values < 0.05 were considered statistically significant.

## Results

### Cohort

A total of 122 examinations formed the analytic cohort. Baseline characteristics and histopathologic outcomes are summarized in Table 1. The distribution of Gleason scores across the cohort reflected a typical tertiary-care referral population.

**Table 1:** Baseline characteristics and histopathologic outcomes

Category	Characteristic	Value (*n* = 122)
Examination Characteristics	Number of Examinations	122
	Age at examination (years), Mean ± SD	66.6 ± 7.3
	PSA (ng/mL), Median [IQR]	8.5 [6.6–12.2]
Histopathology Outcomes	Benign (No PCa)	51 (41.8%)
	GG 1 (Gleason 6)	14 (11.5%)
	csPCa (GG ≥ 2)	57 (46.7%)
AI Findings (Examination Level)	PI-RADS Score 3	13 (10.7%)
	PI-RADS Score 4	42 (34.4%)
	PI-RADS Score 5	36 (29.5%)
	*No Lesion detected*	*31 (25.4%)*
	LoS Score, Median [IQR]	86 [38–98]
AI Findings (Lesion-Level)	Total Number of Lesions Detected	242
	Lesions per Examination, Median [IQR]	1 [0.25-2]
	Lesion Diameter (mm), Median [IQR]	11.0 [7.0–14.0]
	Location: peripheral zone	153 (63.2%)
	Location: transition zone	69 (28.5%)
	Location: central zone	12 (5.0%)
	Location: not determinable	8 (3.3%)

Summary of baseline demographics, histopathological outcomes, and findings from the AI algorithm at both the examination and lesion level. SD = standard deviation, PCa = prostate cancer, GG = Gleason grade group, csPCa = clinically significant prostate cancer, LoS = Level-of-Suspicion, PSA = prostate-specific antigen, IQR = interquartile range

### ROC analysis

**Fig. 1:**
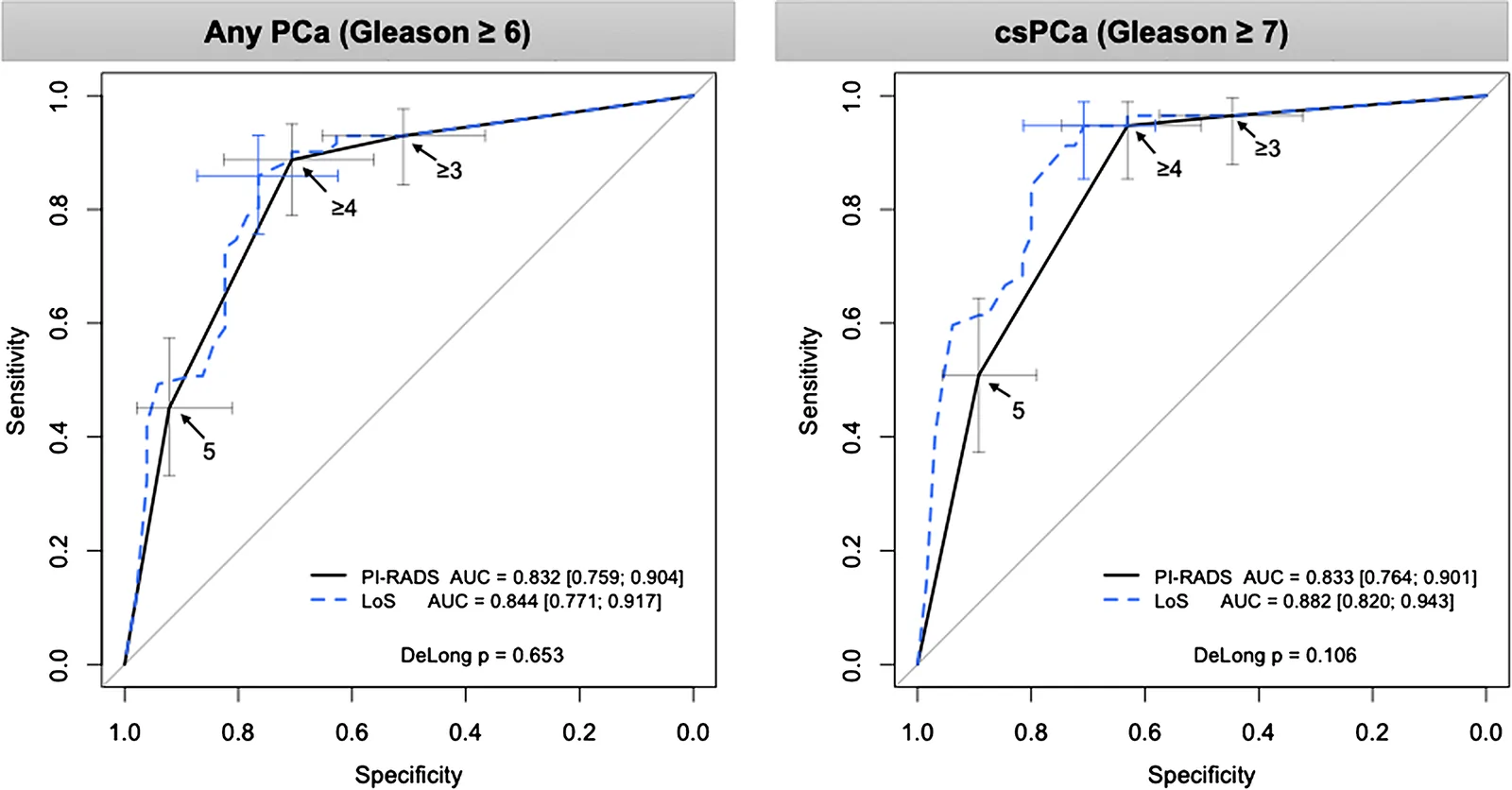
ROC curves for PI-RADS vs. LoS for detection of prostate cancer: Receiver-operating characteristic curves comparing PI-RADS-based classification (black) and the quasi-continuous Level-of-Suspicion (LoS) score (blue) for any prostate cancer (PCa) (left) and clinically significant prostate cancer (csPCa) (right). Error bars show 95% confidence intervals for the PI-RADS cutoffs ≥ 3, ≥4, and 5 (all black), and the LoS score at its optimal cutoff of 81 (blue)

For detecting csPCa (Gleason ≥ 7), the PI-RADS classification achieved an AUC of 0.833 (95% CI: 0.764–0.901). The LoS score as a quasi-continuous classifier achieved a numerically higher AUC of 0.882 (95% CI: 0.820–0.943). DeLong’s test for comparing the two AUCs yielded a p-value of 0.106, indicating a trend favoring the LoS score that did not reach statistical significance (Fig. 1). For detecting any PCa (Gleason ≥ 6), the PI-RADS classification achieved an AUC of 0.832 (95% CI: 0.759–0.904), while the LoS score yielded a numerically higher AUC of 0.844 (95% CI: 0.771–0.917), with this difference not being statistically significant (*p* = 0.653).

### Performance at specific cutoffs

The performance metrics for the PI-RADS classification at conventional cutoffs are shown in Table 2. For csPCa, a PI-RADS cutoff of ≥ 4 preserved high sensitivity (94.7%) with a specificity of 63.1%. The Youden-optimal LoS cutoff was determined to be 81. No examination carried a LoS of exactly 81, the nearest occupied values being 80 and 82. As this threshold was both derived and evaluated in the present cohort, it is reported as an exploratory operating point rather than a validated one. Its performance for both endpoints is detailed in Table 3. For csPCa, this operating point maintained a sensitivity of 94.7% with a specificity of 70.8%, compared with 63.1% at the PI-RADS ≥ 4 threshold. The two decision rules classified every examination identically except for five csPCa-negative examinations with a LoS of exactly 80. In an exact McNemar test on these discordant pairs the difference in specificity did not reach statistical significance (*p* = 0.063). With five discordant examinations the smallest attainable two-sided p value is 0.063, so this comparison cannot reach conventional significance in a cohort of this size. Bootstrap internal validation with 2,000 resamples indicated modest optimism of 1.8% points for sensitivity and 1.7% points for specificity, yielding optimism-corrected estimates of 92.9% and 69.1%. Discrimination was unaffected (corrected AUC 0.882), as no model was fitted and the optimism arises solely from the data-driven choice of threshold. Across resamples, the re-derived threshold ranged from 75.5 to 86.5 (2.5th to 97.5th percentile). The difference in AUC between the two classifiers was 0.049, with a 95% confidence interval ranging from − 0.010 to 0.108, so the data remain compatible with anything from a negligible difference in favour of PI-RADS to an advantage of about one tenth of an AUC unit for the LoS score. Calibration was assessed after mapping the LoS to the probability scale by logistic recalibration. Since the mapping is fitted in the same cohort, the apparent calibration slope and intercept are 1 and 0 by construction and are not informative. The bootstrap-corrected values are a slope of 0.98 and an intercept of -0.01, indicating no material over- or under-spreading of risk. The Brier score was 0.141, against 0.249 for an uninformative model at the observed prevalence.

**Table 2:** AI PI-RADS-based diagnostic performance for any PCa (Gleason ≥ 6) and csPCa (Gleason ≥ 7)

Endpoint	AI PI-RADS Cutoff	Sensitivity (95% CI)	Specificity (95% CI)	PPV (95% CI)	NPV (95% CI)
Gleason ≥ 6	≥ 3	0.930 (0.843–0.977)	0.510 (0.366–0.652)	0.725 (0.622–0.814)	0.839 (0.663–0.945)
	≥ 4	0.887 (0.790–0.950)	0.706 (0.562–0.825)	0.808 (0.703–0.888)	0.818 (0.673–0.918)
	5	0.451 (0.332–0.573)	0.922 (0.811–0.978)	0.889 (0.739–0.969)	0.547 (0.435–0.654)
Gleason ≥ 7	≥ 3	0.965 (0.879–0.996)	0.446 (0.323–0.575)	0.604 (0.496–0.705)	0.935 (0.786–0.992)
	≥ 4	0.947 (0.854–0.989)	0.631 (0.502–0.747)	0.692 (0.578–0.792)	0.932 (0.813–0.986)
	5	0.509 (0.373–0.644)	0.892 (0.791–0.956)	0.806 (0.640–0.918)	0.674 (0.565–0.772)

Sensitivity, specificity, positive predictive value (PPV), and negative predictive value (NPV) at different AI PI-RADS cutoffs with corresponding confidence intervals (CI). PCa = prostate cancer, csPCa = clinically significant prostate cancer

**Table 3:** AI LoS-based diagnostic performance at the Youden-optimal cutoff (81)

Endpoint	Sensitivity (95% CI)	Specificity (95% CI)	PPV (95% CI)	NPV (95% CI)
Gleason ≥ 6	0.859 (0.756–0.930)	0.765 (0.625–0.872)	0.836 (0.730–0.912)	0.796 (0.657–0.898)
Gleason ≥ 7	0.947 (0.854–0.989)	0.708 (0.582–0.814)	0.740 (0.624–0.835)	0.939 (0.831–0.987)

Sensitivity, specificity, positive predictive value (PPV), and negative predictive value (NPV) at the Youden-optimal Level-of-Suspicion (LoS) cutoff of 81 with respective 95% confidence intervals (CI)

### Clinical cases

**Fig. 2:**
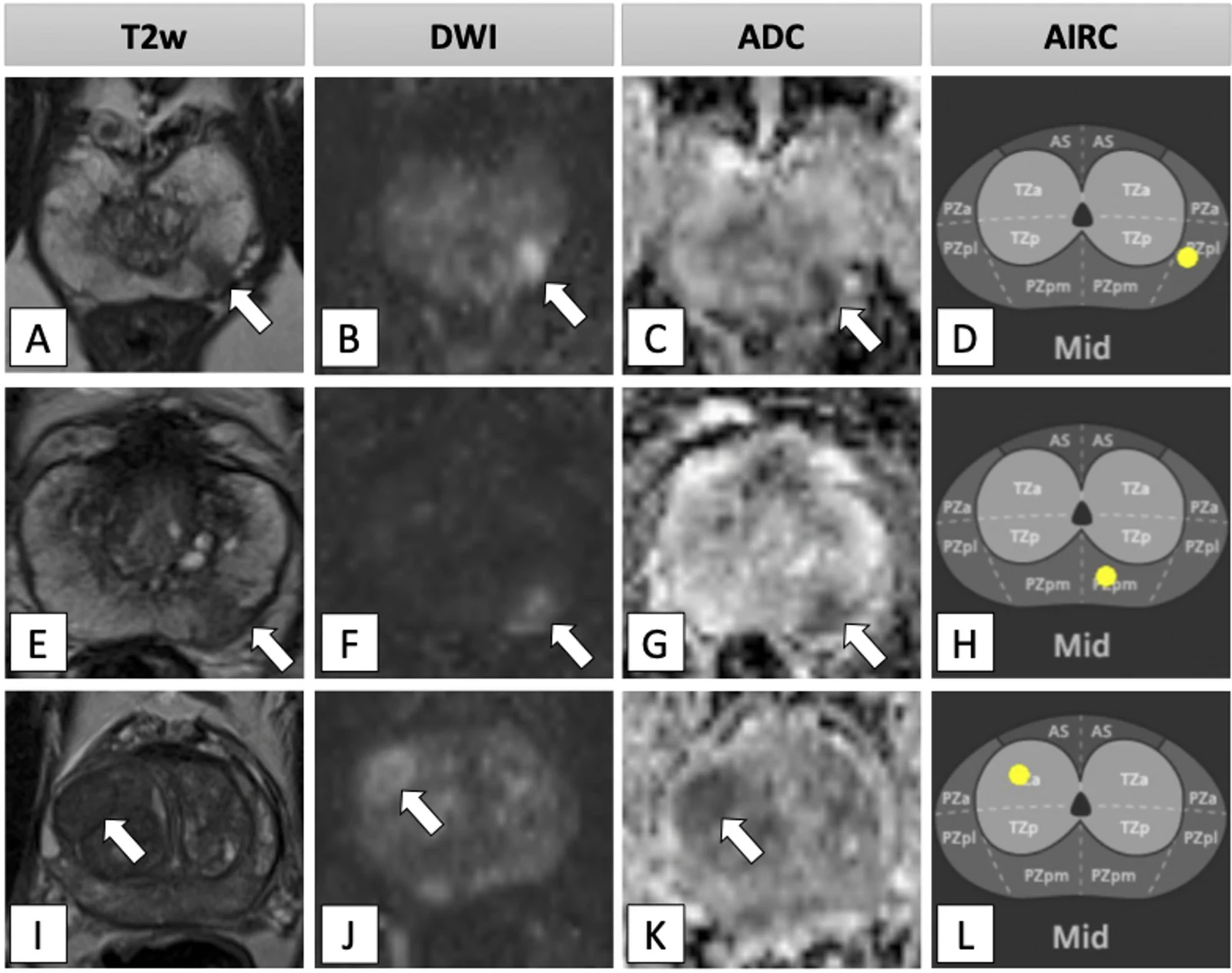
Representative cases illustrating algorithm performance across the diagnostic spectrum: Multiparametric MR images with corresponding AI-Rad Companion (AIRC) outputs for three illustrative cases. Each row displays T2-weighted imaging (T2w; **A, E, I**), diffusion-weighted imaging (DWI; **B, F, J**), apparent diffusion coefficient maps (ADC; **C, G, K**), and the AIRC schematic output with lesion localization (**D, H, L**). Yellow dots indicate algorithm-detected lesions (**D, H, L**). Case 1 (**A-D**): True positive. A 59-year-old man with an algorithm-assigned PI-RADS 4 lesion (LoS 100) in the left posterolateral middle peripheral zone (white arrows). Histopathology confirmed moderately differentiated prostate adenocarcinoma, Gleason score 7 (3 + 4), Gleason Grade Group 2. Case 2 (**E-H**): False positive. A 67-year-old man with an algorithm-assigned PI-RADS 4 lesion (LoS 100) in the left posteromedial middle peripheral zone (white arrows). Despite maximal LoS score, histopathology revealed no evidence of malignancy, illustrating the specificity limitations inherent to imaging-based detection. Case 3 (**I-L**): Sensitivity-favoring classification. A 73-year-old man with a partially encapsulated nodule with restricted diffusion in the right anterior middle transition zone (white arrows). While strict PI-RADS criteria would suggest category 3, the algorithm assigned PI-RADS 4 with an intermediate LoS of 80. Biopsy confirmed acinar adenocarcinoma, Gleason score 6 (3 + 3), Gleason Grade Group 1, so that the elevated suspicion led to the detection of cancer, albeit of a grade below the threshold for clinical significance applied in this study. T2w = T2-weighted; DWI = Diffusion-Weighted Imaging; ADC = Apparent Diffusion Coefficient; AIRC = AI-Rad Companion; LoS = Level-of-Suspicion; PI-RADS = Prostate Imaging Reporting and Data System

To demonstrate the spectrum of algorithm outputs and their clinical correlates, three representative cases are presented in Fig. 2. Case 1 (Fig. 2A-D) illustrates a true positive detection in a 59-year-old man, where the algorithm assigned PI-RADS 4 with a maximal LoS of 100 for a lesion in the left posterolateral middle peripheral zone. Targeted biopsy confirmed a moderately differentiated adenocarcinoma with a maximum Gleason score of 7 (3 + 4), Gleason Grade Group 2, involving up to 90% of individual core. Case 2 (Fig. 2E-H) represents a false positive finding in a 67-year-old man, where the algorithm detected a lesion in the left posteromedial middle peripheral zone with PI-RADS 4 and LoS 100, yet histopathology revealed no malignancy. While formally correct per PI-RADS criteria given the imaging characteristics, this case underscores the inherent specificity limitations of imaging-based cancer detection. Case 3 (Fig. 2I-L) shows a 73-year-old man with a partially encapsulated nodule with restricted diffusion in the right anterior middle transition zone. According to strict PI-RADS v2.1 criteria, this lesion would formally qualify as category 3. However, the algorithm assigned PI-RADS 4 with an intermediate LoS of 80. Histopathology confirmed an acinar adenocarcinoma with Gleason score 6 (3 + 3), Gleason Grade Group 1.

### Descriptive comparison with meta-analytic reference estimates

**Fig. 3:**
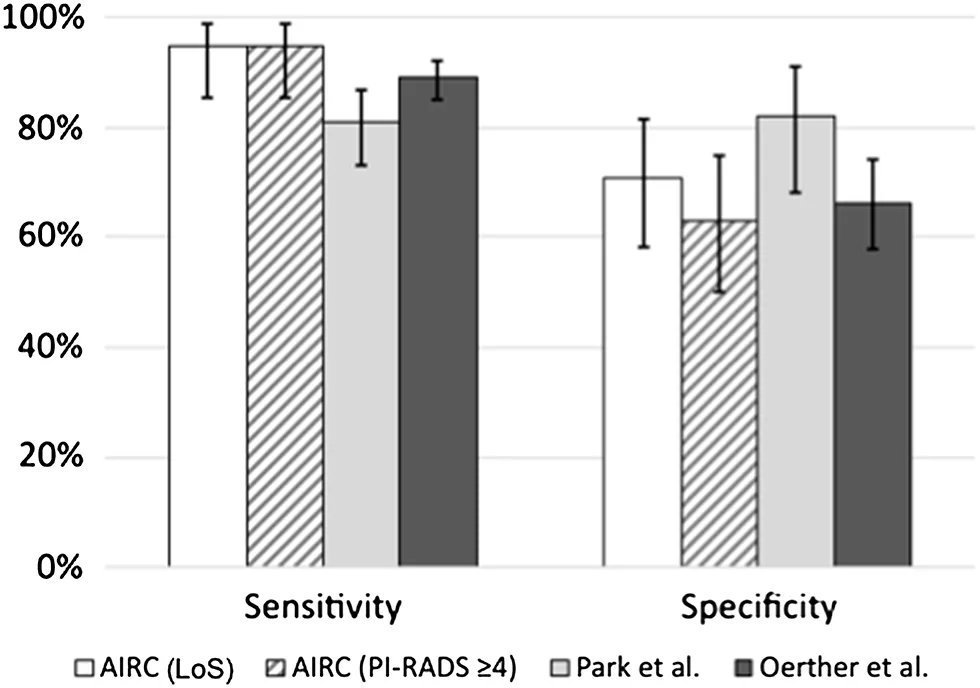
Sensitivity and specificity of the AI-Rad Companion (AIRC) algorithm shown alongside external meta-analytic references from Park et al. [18] and Oerther et al. [19]: Bar chart comparing diagnostic performance for detecting clinically significant prostate cancer (csPCa; Gleason ≥ 7). The AI algorithm was evaluated at its optimal Level-of-Suspicion (LoS) cutoff (white bar) and at the standard PI-RADS ≥ 4 cutoff (column with stripes). Its performance is shown alongside expert reader performance at the PI-RADS ≥ 4 threshold, also given in Table 4, as reported in two meta-analyses: Oerther et al. (dark gray bar; [19]) and Park et al. (light gray bar; [18]). Meta-analytic estimates are shown for context. Error bars indicate 95% confidence intervals. Also see Table 4, which lists all four sets of estimates shown here, and Table 2 for the complete PI-RADS results

To further contextualize the performance of the LoS score at its optimal operating point, we compared its diagnostic metrics for csPCa with those of two large, contemporary meta-analyses of PI-RADS v2.1 performance: Park et al. [18], and the more recent Oerther et al. [19]. Together these analyses summarize more than 14,000 patients, although the underlying primary studies may overlap. As illustrated in Fig. 3, the algorithm’s performance is shown alongside the meta-analytic estimates. At the optimal LoS cutoff (detailed in Table 4), the algorithm achieved a sensitivity of 94.7% (95% CI: 85.4–98.9%). At the standard PI-RADS ≥ 4 cutoff (detailed in Tables 2 and 4), the sensitivity was identical (94.7%). Both results were numerically higher than the sensitivities reported at PI-RADS ≥ 4 by both Park et al. [18] (81%) and Oerther et al. [19] (89%). Regarding specificity, the algorithm’s LoS performance of 70.8% (95% CI: 58.2–81.4%) lay between the two reference estimates. However, at the strict PI-RADS ≥ 4 cutoff, specificity dropped to 63.1%. The corresponding specificities at PI-RADS ≥ 4 are 82% (Park et al. [18]) and 66% (Oerther et al. [19]). These comparisons are descriptive only. The meta-analytic cohorts differ from the present study in patient population, imaging protocol and clinical setting, and no non-inferiority design was pre-specified.

**Table 4:** Descriptive comparison of AI performance at the exploratory optimal LoS cutoff and at the PI-RADS ≥ 4 cutoff with meta-analysis data from Park et al. [18] and Oerther et al. [19] for csPCa

Parameter	AI-Rad Companion (LoS ≥ 81): Estimation (95% CI)	AI-Rad Companion (PI-RADS ≥ 4): Estimation (95% CI)	Park et al., 2021 (PI-RADS ≥ 4): Estimation (95% CI)	Oerther et al., 2024 (PI-RADS ≥ 4): Estimation (95% CI)
Sensitivity	0.947 (0.854–0.989)	0.947 (0.854–0.989)	0.81 (0.73–0.87)	0.89 (0.85–0.92)
Specificity	0.708 (0.582–0.814)	0.631 (0.502–0.747)	0.82 (0.68–0.91)	0.66 (0.58–0.74)

Descriptive comparison of diagnostic metrics for the AI Rad Companion at its exploratory Level-of-Suspicion (LoS) threshold and at the PI-RADS ≥ 4 threshold, alongside pooled data for human readers from two major meta-analyses. Pooled estimates from the meta-analyses are shown for context, for clinically significant prostate cancer (csPCa)

## Discussion

In this clinical evaluation, the AIRC Prostate MR algorithm achieved high diagnostic accuracy for both any PCa and csPCa. Regarding our primary hypothesis, the results showed that the quasi-continuous LoS score yielded numerically higher AUCs compared to the standard ordinal PI-RADS categorization. However, this difference did not reach statistical significance for csPCa (*p* = 0.106). Consequently, the primary hypothesis of superior diagnostic accuracy was not confirmed. The results remain compatible with a quasi-continuous score refining risk stratification beyond fixed ordinal categories, but do not establish such an advantage. The Youden-optimal LoS cutoff of 81 provided an exploratory operating point for csPCa detection, balancing a sensitivity of 94.7% with a specificity of 70.8%. As seen in Fig. 3, the LoS cutoff was associated with a numerically higher specificity than the algorithm’s own PI-RADS ≥ 4 threshold at unchanged sensitivity. This difference did not reach statistical significance either (exact McNemar *p* = 0.063).

The PI-RADS thresholds are familiar but inherently ordinal. In principle, a quasi-continuous LoS score offers an explicit lever to set operating points tailored to clinical needs. In centers prioritizing the rule-out of csPCa (e.g., to reduce unnecessary biopsies), a threshold can be chosen to maximize NPV. The diagnostic accuracy of AI systems for prostate MRI is consistently reported in the recent literature, with AUCs ranging from 0.81 to 0.92, placing our LoS score results well within this range [20–23]. Notably, the Youden-optimal LoS cutoff of 81 lies just above the algorithm’s internal threshold of 80, which separates AI-assigned PI-RADS 3 from PI-RADS 4 or 5. The comparable sensitivity at both cutoffs is therefore expected, while the difference in specificity rests on the examinations at exactly 80, all of which were free of csPCa, rather than on a finer gradation within the high-LoS range. The management of equivocal PI-RADS 3 lesions remains a clinical challenge, and a granular score can help stratify risk within this category, potentially guiding decisions between immediate biopsy and active surveillance [24, 25].

The algorithm’s performance can be placed alongside the meta-analytic data [18, 19], which summarize expert human readers of thousands of mpMRI exams. At the optimal LoS cutoff, the algorithm showed a numerically higher sensitivity than both reference estimates, at a specificity lying between them. This comparison is descriptive only and does not establish superiority over radiologists. Independently of this comparison, a tunable score allows the operating point to be set according to diagnostic priorities.

Strengths of this study include a histopathologic reference standard for every examination and a direct head-to-head ROC comparison of the dual outputs. To our knowledge, this represents the first clinical evaluation of the VA60 version of the commercially available AIRC Prostate MR software. Prior published evaluations were based on earlier algorithm generations. We further placed the algorithm’s performance alongside contemporary benchmarks for human readers reported in the meta-analyses of Park et al. [18] and Oerther et al. [19]. This descriptive comparison places the algorithm alongside those benchmarks rather than ranking it against human readers. The algorithm’s profile prioritizes cancer detection over specificity. This behavior is exemplified by Case 3 (Fig. 2I-L), in which the algorithm assigned a higher PI-RADS category than strict imaging criteria would suggest, yet histopathology confirmed underlying malignancy. Such instances may reflect a design philosophy for clinical routine favoring sensitivity over specificity. Within this cohort, that sensitivity was 94.7%, accompanied by a specificity of 63.1% at the PI-RADS ≥ 4 threshold.

However, limitations must be acknowledged. First, as a retrospective, single-center analysis, generalizability may be limited. Larger, prospective, multi-center studies are required to confirm these findings. Second, although AUC differences favored the LoS score, the difference did not reach statistical significance. With 57 examinations with csPCa and 65 without, the study had limited power to detect a difference of the observed magnitude, which is reflected in the width of the confidence interval of the AUC difference. The absence of a significant difference should therefore not be interpreted as evidence of equivalence. Third, our analysis was at the examination level and did not evaluate lesion-level detection. Fourth, the LoS score is not a probability, so its calibration could only be assessed after logistic recalibration in the same cohort. The resulting calibration is therefore apparent rather than independent, and the score should not be read as a probability of malignancy without recalibration in the target setting. Fifth, the Youden-optimal threshold was derived and evaluated in the same cohort. Although bootstrap internal validation indicated only modest optimism, this threshold requires confirmation in an independent cohort before clinical use. Its higher specificity relative to the PI-RADS ≥ 4 threshold moreover rests on five examinations with a LoS of exactly 80, all of which were free of csPCa, while sensitivity was identical at both thresholds. A margin resting on so few examinations is fragile and may not persist in another cohort. Sixth, although biopsy is the clinical standard, sampling error remains a possibility, as it may not represent the true extent of the disease within the entire gland [26, 27]. Verification was moreover not fully independent of the index test, since targeted cores were directed at lesions visible on MRI. Cancers not visible on MRI therefore entered the reference standard less often, which may bias sensitivity upwards. Prospective studies should validate LoS thresholds across diverse prevalence environments to evaluate cost-utility. It will be crucial to assess reader-in-the-loop performance, measuring AI’s impact on radiologist accuracy and inter-observer agreement [28]. Finally, future work should integrate clinical covariates with the LoS score to build composite risk models. PSA density in particular is a logical candidate for combination with a quasi-continuous AI score. Such composite models have been shown to outperform MRI or clinical data alone, thereby enabling more precise, personalized risk assessment [29–31].

## Conclusion

In this clinical evaluation, the AIRC algorithm for prostate MRI achieved high diagnostic accuracy. For csPCa the quasi-continuous LoS score produced a numerically higher AUC than the conventional PI-RADS classification (0.882 versus 0.833). This difference did not reach statistical significance, so it represents a promising trend rather than a demonstrated advantage. For any PCa the two classifiers performed almost identically (0.844 versus 0.832, *p* = 0.653). The csPCa result nevertheless supports further evaluation of the LoS score as a tunable decision variable complementing fixed PI-RADS thresholds. The exploratory Youden-optimal cutoff of 81 balanced high sensitivity for csPCa with moderate specificity in this cohort and requires prospective validation before clinical adoption. Taken together, these findings support further evaluation of AI-assisted, standardized interpretation in prostate MRI, provided that the operating point is confirmed in an independent cohort.

## Data Availability

The datasets analyzed during the study are available from the corresponding author upon reasonable request.

## Abbreviations

ADC: Apparent diffusion coefficient
AI: Artificial intelligence
AIRC: AI-Rad Companion
AUC: Area under the curve
bpMRI: biparametric magnetic resonance imaging
CI: Confidence interval
csPCa: clinically significant prostate cancer
DWI: Diffusion-weighted imaging
GG: Gleason grade group
LoS: Level-of-Suspicion
mpMRI: Multiparametric magnetic resonance imaging
NPV: Negative predictive value
PCa: Prostate cancer
PI-RADS: Prostate Imaging-Reporting and Data System
PPV: Positive predictive value
PSA: Prostate-specific antigen
T2w: T2-weighted
